# Expression, Purification and Docking Studies on IMe-AGAP, the First Antitumor-analgesic Like Peptide from Iranian Scorpion *Mesobuthus eupeus*

**DOI:** 10.22037/ijpr.2019.15339.13028

**Published:** 2020

**Authors:** Zeinab Dehghan, Hoda Ayat, Ali Mohammad Ahadi

**Affiliations:** *Department of Genetics, School of Science, Shahrekord University, Shahrekord, Iran.*

**Keywords:** IMe-AGAP, Expression, Sodium channels, Analgesic activity, Homology modeling

## Abstract

Scorpion venom contains different toxins with multiple biological functions. IMe-AGAP is the first Analgesic-Antitumor like Peptide (AGAP) isolated from Iranian scorpion *Mesobuthus eupeus*. This peptide is similar to AGAP toxin with high analgesic activity, extracted from Chinese scorpion and inhibits NaV1.8 and NaV1.9 voltage-gated sodium channels involved in the pain pathway. In this study, IMe-AGAP was cloned in a prokaryotic expression vector; expression of toxin in *Escherichia coli* *(E. coli*) was assayed and then purified. In *in-silico* studies, peptide sequence was compared with other scorpion analgesic toxins. The structures of IMe-AGAP and sodium channels were modeled using homology modeling. Structural evaluation and stereo-chemical analysis of modeled structures were performed using RAMPAGE web server Ramachandran plots. Hex Server was used to investigate the interactions between IMe-AGAP and S3-S4 and also S5-S6 segments of NaV1.8 and NaV1.9. Binding energies calculation was used for evaluation of protein docking. Soluble expression of IMe-AGAP in bacteria was investigated by SDS-PAGE analysis. Pure recombinant protein was obtained by Ni-NTA affinity chromatography. The results of three-dimensional structure prediction showed βαββ topology for the toxin that is similar to the conserved structure of α-toxins. Comparison analysis between IMe-AGAP and AGAP toxins exhibited high similarity in homology modeling. Docking analysis demonstrated that IMe-AGAP can interact with NaV1.8 and NaV1.9 domains involved in pain**. **According to the results of homology studies and docking, IMe-AGAP might be a novel potential drug for pain treatment.

## Introduction

Scorpion venom is a rich source of neurotoxic peptides that interact with ion channels (1). Among these peptides, α and β toxins belong to the long-chain neurotoxins and can bind to voltage-gated sodium (NaV) channels. Some of these toxins have analgesic property and are good candidates as pharmaceutical agents (2) . Analgesic toxins interfere with voltage sensors in NaV channels and modify opening channels (3, 4). NaV1.8 and NaV1.9 voltage-gated sodium channels, expressed in ganglia sensory neurons and play important role in pain pathway (5). NaV1.8 channels involved in inflammatory and neuropathic pain and NaV1.9 channels involved in the development of visceral pain associated with acute inflammation (6, 7) . Analgesic toxins interact with two important domain in NaV channels; voltage sensing and pore domains that formed by S1-S4 and S5-S6 helixes respectively. Scorpion alpha toxins keep S3-S4 intracellular loop of domain IV within the cell, resulting in prolonged channel inactivation. Alpha toxins can also bind to channel pore, formed by the extracellular loop between S5 and S6 segments of domain I and IV. This binding slows down the inactivation of the channels (8) . Neuronal damage increases expression of the NaV1.8 and NaV1.9 channels in ganglia sensory cells (9, 10) . Studies have shown that modulating the NaV1.8 and NaV1.9 channels are effective in reducing pain without side effects. Thus, the discovery of these inhibitors promises the development of new analgesic drugs (11, 12).

IMe-AGAP is the first Antitumor-analgesic like peptide isolated from Iranian scorpion *Mesobuthus eupeus* (13). This toxin is similar to BmKAGAP derived from Chinese scorpion *Buthus martensii* and show strong inhibitory effects on somatic and visceral pain. Both peptides have 66 amino acids. Site-directed Mutagenesis studies on BmKAGAP indicate that the Cys16-Cys36 and Cys22-Cys46 disulfide bonds, in addition to the central domain containing glycine 36, arginine 37, tryptophan 57 and asparagine 63 residues are involved in analgesic properties (14). 

Our previous work on the genome sequence of IMe-AGAP showed that this toxin has two exons and one large intron. The sequence of its intron, regulatory elements, and splicing sites are significantly different with other toxins that bind to sodium channels. In the case of peptide sequences, also there are some differences in the IMe-AGAP compared with other analgesic toxins, that can be specific for Iranian subspecies (13) . 

Although these two peptides differ only by three amino acids, sometimes changing one amino acid in toxins can lead to various functions. This is because of the small size of toxins, their highly conserved sequences and very specific recognition sites. Therefore, the main aim of this study is molecular characterization and recombinant production of a novel toxin from Iranian scorpion *Mesobuthus eupeus,* IMe-AGAP. In comparison studies, homology modeling was used to propose the 3D structure of MeI-AGAP and also, the analysis of its interaction with sodium channels was performed to determine the possibility of the analgesic potential of this toxin. 

Regarding the docking results, we tried to clone IMe-AGAP in an expression vector and considered the peptide expression to subsequent functional analysis. Due to the high number of disulfide bonds and toxicity of toxin peptides for the host, the expression of toxin peptides in prokaryotic systems is usually accompanied by various problems (15). So the expression of the appropriate amount of soluble form of the toxin is desirable.

## Experimental

*Gene cloning*

 IMe-AGAP coding fragment has been isolated in previous work (13). The encoding gene was then amplified with tailed primers containing *BamH*I and *Xho*I restriction sites. Purified PCR product was digested with *BamH*I and *Xho*I restriction enzymes (TAKARA, Japan), ligated into multiple cloning sites of pET32b vector and transformed into *E. coli* TOP10 strain. Colony-PCR screening was carried out on recombinant clones by T7 terminator and T7 promoter primers of vector and positive clones were sequenced. 

*Expression of recombinant IMe-AGAP*

 The recombinant vector containing IMe-AGAP was transformed into expression *E. coli* BL21 strain. The bacteria were grown at 37 °C until OD of 0.5 at 600 nm. The cells were induced with 0.1 mM IPTG for 4 h at 28 °C. The bacterial pellet was isolated by centrifuge and resuspend in lysis buffer (50 mM NaH_2_PO_4_, 300 mM NaCl, 10 mM imidazole, pH 8). The sample was sonicated and lysate centrifuged at 10000×g for 20-30 min at 4 °C. The supernatant containing soluble protein and pellet with insoluble matter along with non-induced samples were loaded in SDS-PAGE, stained with comassie brilliant blue (CBB) R250 (Sigma, USA). 

*Purification of IMe-AGAP protein*

About 6 mL supernatant of lysate was applied to Ni-NTA column (Qiagen, Hilden, Germany). Following twice washing with 4 mL wash buffer (50 mM NaH_2_PO_4_, 300 mM NaCl, 20 mM imidazole, pH 8), the protein was eluted twice with 0.5 mL of elution buffer (50 mM NaH_2_PO_4_, 300 mM NaCl, 250 and 500 mM imidazole, pH 8). All the fractions were collected and analyzed by 12% SDS-PAGE.

*Bioinformatics studies*

*Study of protein sequence*

The analgesic toxins and sodium channel sequences were retrieved from biological databases NCBI (http://www.ncbi.nlm.nih.gov/) and uiprot (http://www.uniprot.org/). Access numbers (ID) of the NaV1.8 and NaV1.9 sodium channels and scorpion toxins in NCBI and uniprot database are listed in Tables 1 and 2. CLC main work bench5 software was used to compare and find similar structural motif of IMe-AGAP protein sequence with α and β analgesic toxins. Phylogenetic studies were performed to classify scorpion toxins and evolutionary relationship by clustalw (https://www.genome.jp/tools-bin/clustalw) (16, 17).

*3-D structure prediction and structural homology studies*

 Toxin structure prediction can determine the structural and functional relationship of toxins, functional amino acids, and their catalytic status. Disulfide bonds are important factors in toxin folding and function. DIANNA server was used to determine disulfide bonds between the cysteine amino acids in toxin. Prediction of three-dimensional structures of toxins and sodium channels was performed by phyre2 (v2.0) (www.sbg.bio.ic.ac.uk/~phyre2/) and CPHmodel (3.2) (www.cbs.dtu.dk/services/CPHmodels/) web servers. The evaluation of toxin modeling structure was accomplished using RAMPAGE web server Ramachandran plots. Yasara software was used to study IMe-AGAP and α-toxins structural homology, based on similarity percent and RMSD. 

*Interaction model of IMe-AGAP and sodium channels*

The docking analysis of IMe-AGAP and IV domain of NaV1.8 as well as NaV1.9 sodium channels was carried out by Hex (http://hex.loria.fr/) web server. Two protein structures in PDB format are essential for uploading by this server. In hex web server, IV domain of NaV1.8 and NaV1.9 sodium channels were treated as receptors and IMe-AGAP was treated as the ligand. The parameters used for the docking process via Hex Server were: range of receptor and ligand rotation angle, 180 degrees Z around; step size of the receptor and ligand, 7.5 degrees; the output of docking, 100 number; twist range of receptor and ligand, 360 degrees; distance range of ligand and receptor, 40 ^0Å^. Yasara (v11.11.2), pymol, and VMD (v1.9.1) softwares were used to compute the total energy of each complex model, H-bond and hydrophobic interactions and residues involved in interactions. These softwares are molecular graphics programs intended for the structural visualization of proteins. 

## Results

*Cloning, expression, and purification of IMe-AGAP*

**Figure 1:**
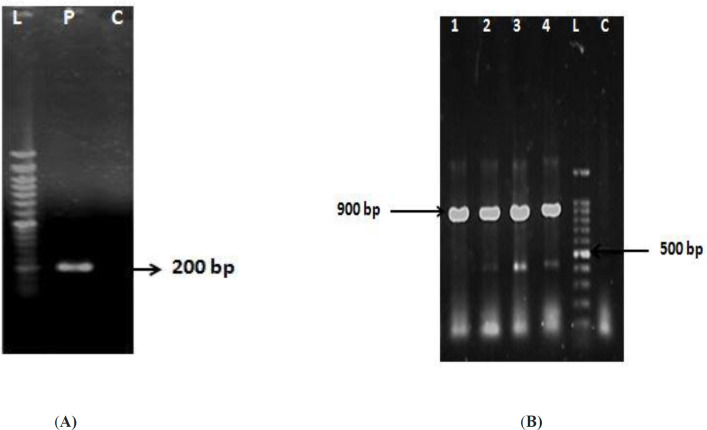
(A) Amplification of IMe-AGAP gene with product size of ~ 200 bp is seen in P line. L: 50 bp DNA ladder. (B) Results of colony-PCR with T7 promoter primer of vector and reverse primer of gene. L: 100 bp DNA ladder, 1-4: positive clones with size band ~ 900 bp, C: Original vector

**Figure 2:**
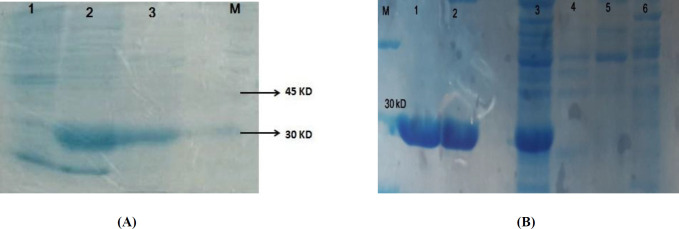
(A) Expression of IMe-AGAP protein in *E. coli* Bl21 strain. Lane 1 is pre-induction culture, lane 2: post- induction soluble form and lane 3 is insoluble fraction of protein extraction. A band around 30 kD is seen in 2 and 3 lines. (B) Purification of IMe-AGAP protein with Ni-NTA column, 1 and 2 are purified elutions. 3 is pre-purification sample. 4 and 5 washing buffer and 6 is sample after purification

IMe-AGAP gene was amplified and cloned in the corresponding site of pET32b (Figure 1A). The recombinant vector was verified by PCR (Figure 1B) and sequencing. Expression of soluble recombinant protein was analyzed in 12% SDS-PAGE. In comparison with the induced cells and non-induced cells, one band about 26 kD was seen that was calculated theoretically (total weight of recombinant protein~7.3 kD and thioredoxin as a fusion part of vector~ 18.5 kD). Most of the recombinant protein was detected in soluble fraction (Figure 2A). The recombinant protein was purified by Ni-NTA column that the existence of the His-tag on IMe-AGAP was indicated (Figure 2B).

*Comparison of toxin protein sequences*

**Figure 3:**
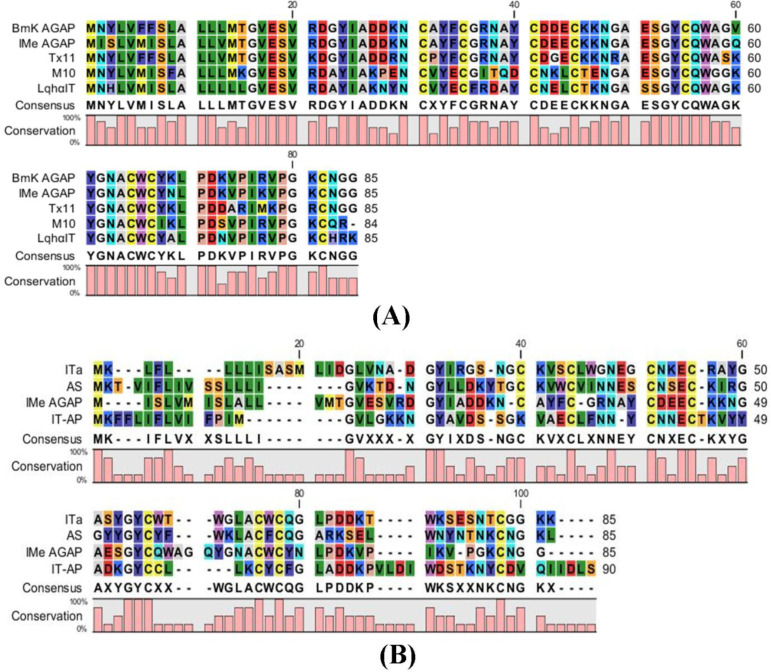
Homology analysis of toxin protein sequence performed by CLC Main work bench 5 software. (A) Comparison of IMe-AGAP and alpha toxins protein sequence. (B) Comparison of IMe-AGAP and beta toxin protein sequences

Comparative studies of peptide sequences were done to find the structure and function of IMe-AGAP. Comparison of IMe-AGAP protein sequence with α-toxins (BmKAGAP, M10, TX11, and LqhαIT) and β-toxins (BmKIT-AP, BmKITa and BmKAS) was performed by CLC software and showed the high similarity of IMe-AGAP with alpha toxins compared with beta toxins. Alpha toxins contain 60-72 amino acids in mature form. The SignalP 4.1 server revealed that the signal peptides of toxins are about 18-22 amino acids. The similarity between whole IMe-AGAP and BmKAGAP (along with signal peptide) sequence protein was calculated 92%, although the similarity of the mature peptide sequences is about 96%. Four residues of signal peptides [1-19] are different in BmKAGAP and IMe-AGAP which are respectively; Asp/Ile [2], Tyr/Ser [3], Phe/Met [6], and Phe/Ile [7]. The regions encoding the mature peptide of two toxins are different in three amino acids. Polar negatively charged aspartic acid [43], nonpolar valine [60], and polar positively charged lysine [69] in the BmKAGAP peptide changed by polar negatively charged glutamic acid [43], polar glutamine [60], and polar asparagine [69] amino acids in the IMe-AGAP peptide (Figure 3).

Disulfide bonds in cysteine amino acids play an important role in toxin folding and are conserved in alpha and beta toxins. Results of DIANNA server showed that IMe-AGAP disulfide bonds are similar to BmKAGAP peptide. The disulfide bonds are formed between 12-63, 16-36, 22-46, and 26-48 cysteine ​​residues (Table 3). 

**Figure 4:**
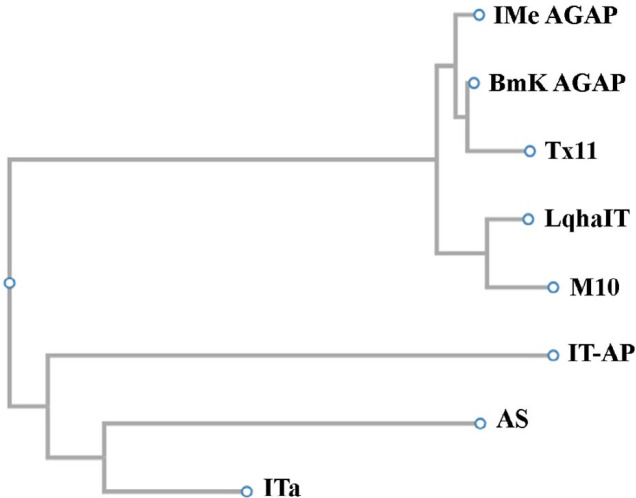
Results of phylogenetic studies that have been achieved by Clustal w server

Phylogenetic studies between some α-toxins showed that IMe-AGAP from *Mesobuthus eupeus* scorpion and BmKAGAP toxin from *Buthus martensii* are in a branch of the evolutionary tree. ITα and AS toxins both isolated from *Mesobuthus martensii* scorpion are very similar, these toxins were created from an ancestral gene. M10 and LqhαIT which are isolated from *Mesobuthus martensii* and *Leiurus quinquestriatus hebraeus* scorpion respectively are a branch of the evolutionary tree so these toxins can be derived from an ancestral gene (Figure 4). These results are consistent with taxonomy data in NCBI website.

*3D structure determination and comparison analysis*

The three-dimensional structures of toxins were determined by CPHmodel and phyre2 web servers and showed that both alpha and beta toxins bind to Na^+^ channel despite different amino acids in their structure. These toxins have a protected Knottin domain. Knottin domain is formed when the III-VI disulfide bridge passes between disulfide bonds of I-IV and II-V cysteines. Structural evaluation of modeled structures was performed by using RAMPAGE web server Ramachandran plots (Table 4). 

**Figure 5:**
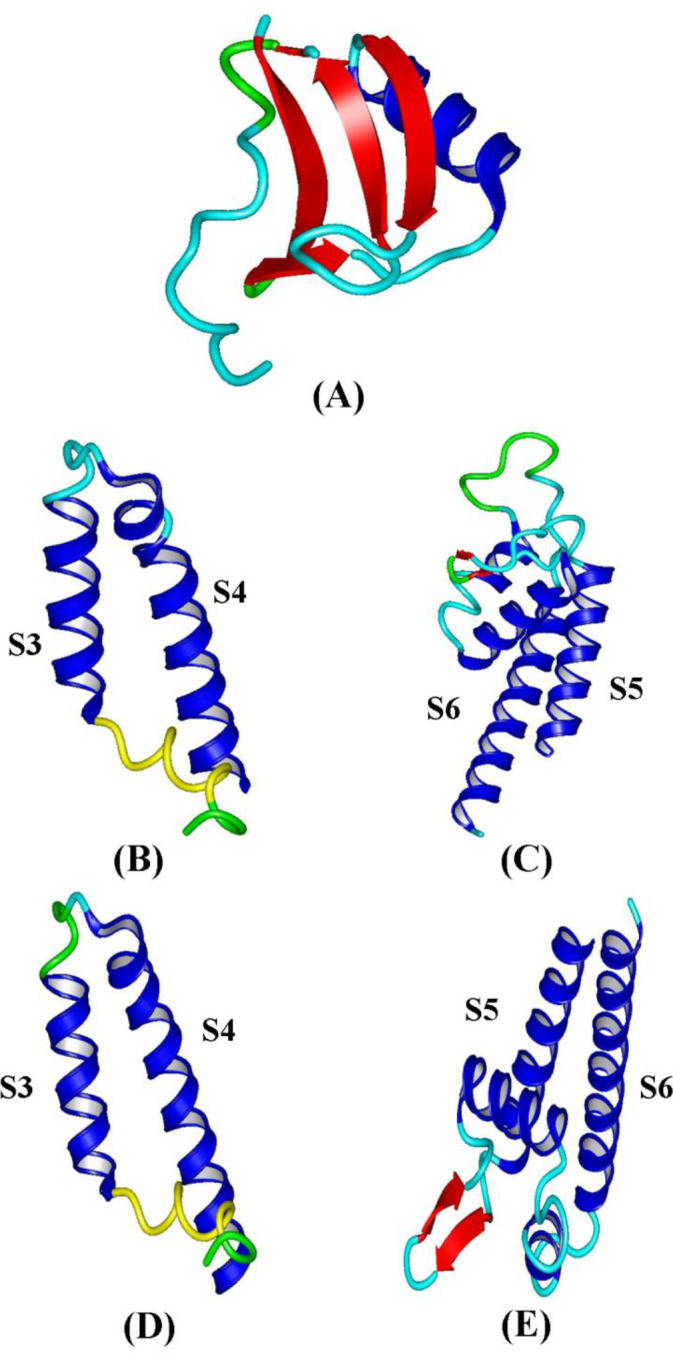
The three-dimensional structures obtained by Phyre2 and CPHmodel. (A) Analgesic toxins. (B) (S3-S4) IV domain Na_V_1.9. (C) (S5-S6) IV domain Na_V_1.9. (D) (S3-S4) IV domain Na_V_1.8. (E) (S5-S6) IV domain Na_V_1.8

Phyre2 web server was used to the prediction of the structure of S3-S4 and S5-S6 segments in NaV1.8 and NaV1.9 sodium channels. S3-S4 segment has two transmembrane alpha-helices and one extracellular short loop. S5-S6 segment has also two transmembrane alpha-helices, one extracellular alpha-helix with two short loops. Toxins and sodium channels structure is shown in Figure 5.

The results of IMe-AGAP and alpha toxins structural comparison revealed that the IMe-AGAP has the most similarity (93/85%) with BmKAGAP and the lowest similarity (67.74%) with BmKM10. The distance between structures is in positions Glu [41]/IMe-AGAP and Val [41]/BmKAGAP, Ile [18]/IMe-AGAP and Gly [39]/BmKM10, Glu [41] and Pro [56]/IMe-AGAP and Lys [41] and Arg [56]/BmKTX11, Ala [39] and Gly [65,66] /IMe-AGAP and Trp [38] ,Arg [65] and Lys [66] /Lqα-IT. These results are shown in Table 5.

*Docking analysis*

**Figure 6:**
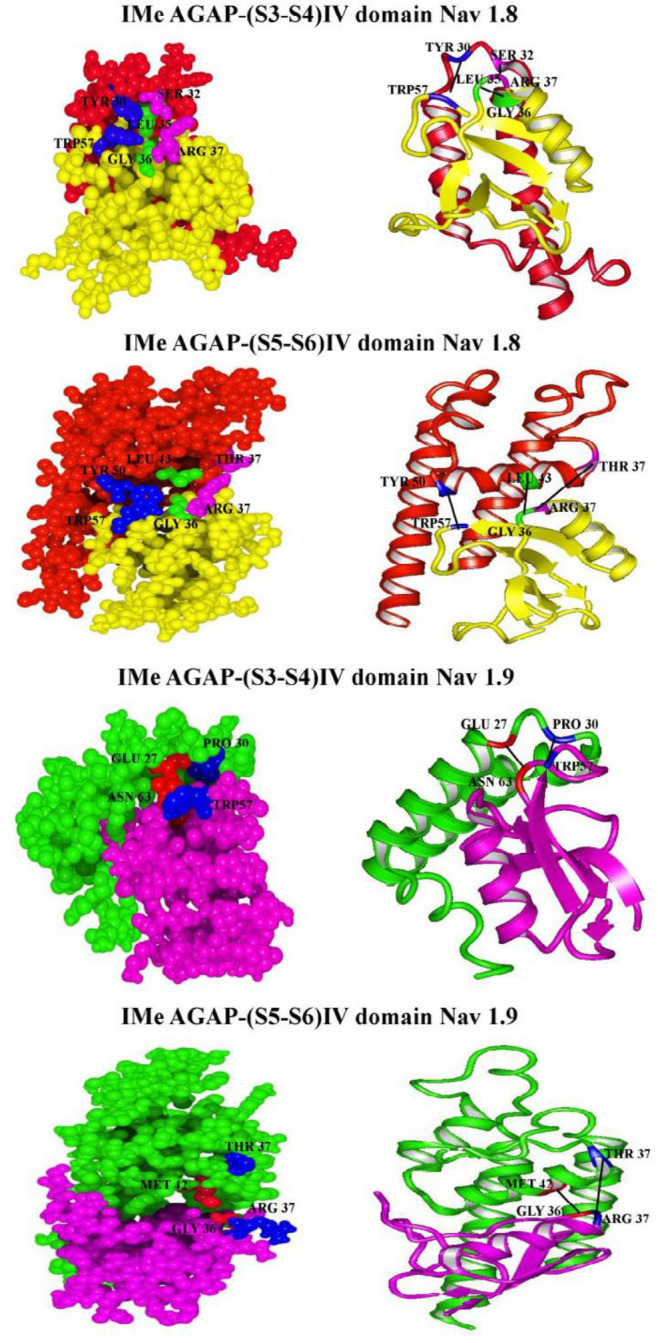
Ribbon and ball models of IMe-AGAP-sodium channels IV domain complexes. Whole backbones of complexes are shown. The amino acid residues involved in interaction between IMe-AGAP - sodium channels IV are indicated

 To investigate the interactions between IMe-AGAP and IV domain of sodium channels, we used hex server (Figure 6). The lowest free energy of interaction and also the best tendency interaction to the ligand was obtained. Table 6 shows residues involved in hydrogen bonds and hydrophobic interactions between IMe-AGAP and IV domain, and also interaction between total energies and bond lengths. The docking results between IMe-AGAP and Na1.8 showed that two hydrogen bonds, Trp [57] – Tyr [50] and Arg [37] – Thr [37], in addition to one hydrophobic interaction, Gly [36] – Leu [43] are formed in IMe-AGAP-IVS5-S6 complex. The binding energy of these interactions is -847.4 j/mol. The same results were seen between IVS3-S4 domain of channel and toxin. In IMe-AGAP-IVS3-S4 domain complex, two hydrogen bonds, Tyr [30] - Trp [57] and Ser [32] - Arg [37], in addition to one hydrophobic interaction, Leu [35] - Gly [36], are formed. These interactions have a binding energy about -943.1 j/mol that is stronger than S5-S6 domain and IMe-AGAP interactions.

Interaction analysis of Na1.9 channel and IMe-AGAP only showed two hydrogen bonds between Glu27 and Pro30 in S3-S4 domain with Asn 63 and Trp 57 in toxin, respectively. The binding energy of these interactions is about -431.8 j/mol. In IMe-AGAP-IVS5-S6 complex, one hydrogen bond, Arg 37 - Thr 37 and one hydrophobic interaction, Gly 36 - Met 42 are formed, that have the binding energy about -518 j/mol. Therefore, in Na1.9 channel, the interaction of S5-S6 domain with toxin is stronger than S3-S4 domain.

**Table 1:** Access number of Na_V_1.8 and Na_V_1.9 sodium channels in uniprot database

**number access**	**sodium channels**
Q9Y5Y9	Sodium channel protein type 10 subunit alpha (Na_V_1.8)
Q9UI33	Sodium channel protein type 11 subunit alpha (Na_V_1.9)

**Table 2:** Access number of toxins in NCBI and uniprot database

**Toxins**	**Toxins group**	**number access**	**Species**
IMe-AGAP	α- toxin	AJF23104.1	*Mesobuthus eupeus*
BmK-AGAP	α- toxin	Q95P69	*Mesobuthus martensii*
BmK-AS	β-toxin	Q9UAC9	*Mesobuthus martensii*
BmK-M10	α- toxin	O61705	*Mesobuthus martensii*
BmK-ITα	β-toxin	Q9XY87	*Mesobuthus martensii*
BmK-IT-AP	β-toxin	O77091	*Mesobuthus martensii*
BmK-TX11	α- toxin	55741121	*Mesobuthus martensii*
LqhαIT	α- toxin	134374	*Leiurus quinquestriatus hebraeus*

**Table 3:** Disulfide bonds obtained by DIANNA server in IMe-AGAP

**Cysteine position**	**Distance**	**Bond**	**Score**
12 - 63	51	ADDKNCAYFCG - KVPGKCNGGXX	0.99806
16 - 36	20	NCAYFCGRNAY - AESGYCQWAGQ	0.99911
22 - 46	24	GRNAYCDEECK - QYGNACWCYNL	0.99728
26 - 48	22	YCDEECKKNGA - GNACWCYNLPD	0.9952

**Table 4.** Ramachandran plot calculations for 3D model of toxins

**Toxins**	**Amino acid in most favored regions (%)**	**Amino acid in allowed regions (%)**	**Amino acid in outlier regions (%)**
IMe-AGAP	98.4	1.6	0.0
BmKAGAP	90.6	6.2	3.1
TX11	85.9	10.9	3.1
M10	88.7	4.8	6.5
LqhαIT	88.7	9.7	1.6
AS	88.1	10.2	1.7
ITα	96.6	3.4	0.0
IT-AP	95.7	4.3	0.0

**Table 5:** Comparative study of IMe-AGAP and α-toxins structures, RMSD and similarity% were performed through yasara software

**similarity (%)**	**RMSD**	
93.85	0.974 A^0^	IMe-AGAP – BmKAGAP
78.46	0.959 A^0^	IMe-AGAP - TX11
72.31	1.495 A^0^	IMe-AGAP - Lqα-IT
67.74	1.149 A^0^	IMe-AGAP - M10

**Table 6:** Docking results: total binding energies, interactive residues by hydrogen and hydrophobic bonds, bond distances between MeI- AGAP and sodium channels IV domain complexes

	**Energy (j/mol)**	**Amino acids involved in interaction**	**Bond distance**	**Bond type**
IMe-AGAP-(S3-S4) IV domain 1.8	- 943.1	Na_V_ 1.8 – AGAP Tyr 30 – Trp 57 Ser 32 – Arg 37 Leu 35 – Gly 36	9 A^0^ 9 A^0^ 12.5 A^0^	Hydrogen (O-N) Hydrogen (O-N) Hydrophobic (C-H)
IMe-AGAP- (S5-S6)IV domain 1.8	- 847.4	Na_V_1.8 – AGAP Thr 50 – Trp 57 Leu 43 – Gly 36 Thr 37 – Arg 37	7.3 A^0^ 9.8 A^0^ 14.9 A^0^	Hydrogen (O-N) Hydrophobic (C-H) Hydrogen (O-N)
IMe-AGAP- (S3-S4) IV domain 1.9	- 431.8	Na_V_1.9 – AGAP Glu 27 – Asn 63 Pro 30 – Trp 57	6.52 A^0^ 8.22 A^0^	Hydrogen (O-N) Hydrogen(O-N)
IMe-AGAP- (S5-S6) IV domain 1.9	- 518.3	Na_V_1.9 - AGAP Thr 37 – Arg 37 Met 42 – Gly 36	12.51 A^0^ 7.78 A^0^	Hydrogen (O-N) Hydrophobic (S-H)

## Discussion

Due to the limited amount of toxins in scorpion crude venom and severe and high-cost purification conditions, cloning of toxin genes and expression techniques have been replaced. Among different expression systems, expression in *E. coli* is the most feasible and disposal. However, the high numbers of disulfide bonds, usually result in the insoluble form of recombinant toxins in bacteria. Since inclusion body was formed with the high level expression of BMkAGAP in E. coli, Cao *et al.* generated bioactive form of the peptide for further application by *in-vitro* refolding analysis (18, 19). To enhance soluble expression of rBMkAGAP, other approaches have also been used such as the expression of the peptide in plants or SUMO fusion technology and co-expression of AGAP with thioredoxin in *E. coli *(20-22). In this study, we cloned IMe-AGAP gene into a pET32b vector that contains thioredoxin as a fusion form with the cloned peptide. It promotes the correct formation of disulfide bonds in toxin and also increases the solubility of the peptide in bacteria several times. In this study, an analgesic toxin from an Iranain scorpion was cloned and successfully expressed and purified, for the first time. Herein, we obtained sufficient amount of IMe-AGAP peptide as soluble protein for functional studies in the future without refolding steps.

 The *in-silico* studies on IMe-AGAP showed this peptide can be developed as an analgesic agent for treatment of pain. Multiple sequence alignment of IMe-AGAP with some scorpion toxins revealed high sequence homology of this toxin with scorpion α-toxins such as, BmK AGAP (96%). Because there is no clear relationship between toxin sequences and their analgesic activity, the three-dimensional structure of IMe-AGAP was obtained from phyre2. Scorpion α-toxins that are sodium channel inhibitor showed the conservative structural core with a βαββ conformation and four disulfide bridges (23). IMe-AGAP is a typical alpha-scorpion toxin that has a common βαββ with four disulfide bridges (Cys 12–Cys 63, Cys 16–Cys 36, Cys 22–Cys 46, and Cys 26-Cys 48). Ramachandran plots analysis of these models determined the most residues that were in the most favored region. 

The N-terminus, the C-terminus and some conserved aromatic residues Tyr 5, 14, 21, 35, 42 and Trp 38, 47 are very important in the structure and function of α-toxins. Some positively charged residues especially in C-terminus form a putative area that is conserved in these toxins and is related to the receptor binding sites. Lys 8, Arg 18, Lys 62, and Arg 64 form electrostatic potential that may interact directly with the corresponding receptor site. In IMe-AGAP sequence and structure, these residues are conserved that could show the analgesic property of this toxin. 

BMkAGAP displayed strong analgesic effects in whole animal tests. This toxin and IMe-AGAP mature peptide sequence shows high homology and differing in only three amino acids; Asp 43 to Glu, Val 60 to Gln, and Lys 69 to Asn, respectively. Structural homology analysis determined by yasara software showed that the IMe-AGAP has the most similarity (93/85% and 0.974 A^0^ RMSD) with BmKAGAP that means these substitutions may have no significant effect on the structure of peptides and probably on IMe-AGAP activity. 

Finally, docking studies were carried out to determine the interaction of IMe-AGAP and Na channels. The low value, negative binding energy, and number of H2 bonds show most appropriate binding between protein and ligand molecules (24, 25). More stable complex is selected according to the total energy and the number of interactive residues. Selected sodium channels IV domain and IMe-AGAP complex are more stable according to the total energy and the number of interactive residues. There are 4 hydrogen bonds and 2 hydrophobic interactions, between IMe-AGAP and IV domain of Na1.8, also there are 3 hydrogen bonds and 1 hydrophobic interaction between IMe-AGAP, and IV domain of Na1.9. Mutagenesis studies of Ma and coworker exhibited that Glycine 36, Arginine 37, Tryptophan 57, and Aspargine 63 in BmKAGAP show analgesic properties (26) . Our obtained results of IMe-AGAP and sodium channels docking, mentioned in the results section, were in agreement with the previous finding. Due to this high similarity between IMe-AGAP and BmKAGAP structure and docking, we hope that this toxin also can be a good candidate for the treatment of pain. Of course, a better understanding of these toxins could be obtained by functional experiments in the future. 
